# Phase II trial of upfront bevacizumab and temozolomide for unresectable or multifocal glioblastoma

**DOI:** 10.1002/cam4.58

**Published:** 2013-01-24

**Authors:** Emil Lou, Katherine B Peters, Ashley L Sumrall, Annick Desjardins, David A Reardon, Eric S Lipp, James E Herndon, April Coan, Leighann Bailey, Scott Turner, Henry S Friedman, James J Vredenburgh

**Affiliations:** 1 The Preston Robert Tisch Brain Tumor Center at Duke, Department of Surgery, Duke University Medical CenterDurham North Carolina; 2 The Preston Robert Tisch Brain Tumor Center at Duke, Department of Medicine, Duke University Medical Center DurhamNorth Carolina; 3 Center for Neuro-Oncology, Dana-Farber Cancer InstituteBoston, Massachusetts; 4 The Preston Robert Tisch Brain Tumor Center at Duke, Department of Surgery, Duke University Medical Center DurhamNorth Carolina; 5 Cancer Center, Department of Biostatistics, Duke University Medical CenterDurham, North Carolina; 6 The Preston Robert Tisch Brain Tumor Center at Duke, Department of Surgery and Pediatrics, Duke University Medical CenterDurham, North Carolina

**Keywords:** Bevacizumab, glioblastoma, neoadjuvant therapy, temozolomide, unresectable, upfront

## Abstract

Patients with unresectable glioblastomas have a poor prognosis, with median survival of 6–10 months. We conducted a phase II trial of upfront 5-day temozolomide (TMZ) and bevacizumab (BV) in patients with newly diagnosed unresectable or multifocal glioblastoma. Patients received up to four cycles of TMZ at 200 mg/m^2^ on days 1–5, and BV at 10 mg/kg on days 1 and 15 of a 28-day cycle. Brain magnetic resonance imaging (MRI) was performed monthly. Therapy was continued as long as there was no tumor progression, grade 4 nonhematologic toxicity, or recurrent grade 4 hematologic toxicity after dose reduction. The primary end point was best tumor response as measured on MRI. Forty-one patients were accrued over 12 months; 39 had a full set of MRI scans available for evaluation. Assessment for best radiographic responses was as follows: partial responses in 24.4%, stable disease in 68.3%, and progressive disease in 2.4%. Treatment-related toxicities included seven grade 4 toxicities and one grade 5 toxicity (myocardial infarction). From this study, it was concluded that an upfront regimen of TMZ and BV for unresectable glioblastoma was well tolerated and provided a significant level of disease stabilization. Therapeutic toxicities were consistent with those seen in the adjuvant setting using these agents. The upfront approach to treatment of glioblastoma in the unresectable population warrants further investigation in randomized controlled phase III trials.

## Introduction

Glioblastomas (GB) are associated with significant morbidity and a dismal prognosis. Median overall survival (OS) ranges between 8 and 16 months 1. The optimal treatment of GB is surgical resection, followed by concurrent chemoradiation using temozolomide (TMZ) therapy 1. Adjuvant therapy consists of 6–12 months of standard 5-day TMZ at 150–200 mg/m^2^ per 28-day cycle. In a study by Stupp et al. 1, adjuvant chemoradiation with TMZ was shown to improve outcomes in patients with newly diagnosed GB; however, the median survival in the subpopulation of patients with unresectable disease was 9.4 months compared with 15.8 months in patients with resected disease (*P* < 0.001). Moreover, for patients with unresectable disease, the median survival was similar for radiation therapy (7.9 months) and chemoradiation with TMZ (9.4 months). These data suggest that prognosis is better for patients who undergo gross total resection than for patients who undergo subtotal resection or who cannot undergo surgery due to tumor location 2–5.

Despite this poor prognosis, patients with multifocal or single-lesion unresectable GB tumors have no standard treatment options or approaches aside from standard chemoradiation. Neoadjuvant chemotherapy has been used to treat several solid tumor malignancies that are difficult to resect or treat upon initial presentation, including breast 6,7, pancreatic 8, metastatic colorectal 9, locally invasive rectal 10, and cervical cancers 11. The rationale underlying neoadjuvant therapy is to downsize the tumor prior to surgical resection or debulking. Additionally, this approach allows for assessment of chemosensitivity prior to surgical removal and adjuvant treatment. In the treatment of GB, upfront therapy with chemotherapy for a specified amount of time prior to chemoradiation is similar to the neoadjuvant approach in systemic malignancies.

For patients with unresectable GB tumors, inhibition of vascular endothelial growth factor (VEGF) may improve survival and disease-related morbidity. In 2009, bevacizumab (BV) was approved by the Food and Drug Administration (FDA) for treatment of recurrent GB 12. Our group recently published a phase II trial of 75 patients with resected disease for whom BV was added to concurrent chemoradiation in an adjuvant setting; BV was given in addition to 5-day TMZ and irinotecan, a topoisomerase I inhibitor 13. This regimen had moderate toxicity, and resulted in a median progression-free survival (PFS) of 14.2 months and median OS of 21.1 months.

However, a neoadjuvant or upfront approach in which these patients are pretreated with standard TMZ and BV prior to combined chemoradiation has not yet been assessed. We hypothesized that the combination of TMZ and BV would demonstrate significant biologic activity as assessed by radiologic response of unresectable GB tumors. On the basis of the promising results of our prior study, we proposed that treating unresectable GB with TMZ/BV prior to, rather than following, chemoradiation would improve outcomes as well as tumor- and treatment-related morbidity. To test this hypothesis, we conducted a phase II, single-institution trial of upfront TMZ/BV in patients with single-lesion or multifocal unresectable GB. All patients were treated for four monthly cycles, barring significant adverse events (AE) or progressive disease (PD), prior to chemoradiation.

## Patients and Methods

### Patient eligibility criteria

Patients with newly diagnosed unresectable single or multifocal GB were enrolled into this single-institution, single-arm, phase II trial if they were adult patients greater than 18 years of age. Patients were deemed unresectable if their tumors were not deemed resectable by neurosurgeons at our institution, following magnetic resonance imaging (MRI) review. Reasons for deeming tumors unresectable included tumor location not amenable to surgery, and/or multifocal disease. The protocol was written to include both unresectable (biopsy only) and subtotal resection patients as eligible for enrollment. Our institution sees a relatively large number of both of these populations. However, due to interest in trial enrollment and large patient volume, we were able to fill all 41 spots with patients with unresected, biopsy-only GB. Criteria for enrollment also included Karnofsky performance score (KPS) ≥60%; no prior treatment, including radiation or chemotherapy; at least 1 week from closed biopsy; and no evidence of grade 2 or higher central nervous system (CNS) hemorrhage. Patients with a history of hypertension could be enrolled, but only if their hypertension was stable and managed, with blood pressure demonstrated to be consistently below 140/90. Patients on therapeutic anticoagulation were excluded; however, patients who developed thromboembolic complications while on study were initiated on anticoagulation therapy and remained on study. This study was conducted at Duke University Medical Center, Durham, North Carolina, and approved by the Duke University Institutional Review Board (IRB). All patients provided written informed consent.

### Treatment plan

All patients who enrolled received TMZ at a dose of 200 mg/m^2^ on days 1 through 5 of each 28-day cycle and BV at a dose of 10 mg/kg on days 1 and 15 of each cycle. The study was designed to enroll 41 patients who met all eligibility criteria, with an intention to treat for up to four full cycles prior to standard combined chemoradiation. All patients were required to receive the first infusion of BV at Duke University Medical Center; subsequent infusions were administered by a patient's local oncologist when feasible. Patients were regularly monitored for treatment- and disease-related morbidity. Infusions of BV were given at our institution when possible. However, due to the large geographic distribution of patients enrolled on study, allowances were made for patients to receive interim infusions closer to home by board-certified medical oncologists. We received and reviewed the notes from all outside clinic visits and infusions every 2 weeks to document BV administration and any noted toxicities per CTCAE v.3 criteria. These data were included in ongoing and final assessments of AE. The FDA approved and provided guidance for a specific program for monitoring BV infusions administered by outside oncologists. A clinical trial coordinator (L. B.) called the local oncologists regularly. In addition, the principal investigator of this study (J. J. V.) personally reviewed all laboratory values and vital signs before administration of each BV infusion given while on study.

Points of assessment included blood pressure monitoring every 2 weeks, complete blood count (CBC) with automated differential on days 21 and 28 of each cycle, comprehensive metabolic panel every 4 weeks, and urinalysis or protein-to-creatinine ratio on spot urinalysis every 4 weeks. Each patient was evaluated with a complete physical examination including comprehensive neurologic examination and toxicity assessment every 4 weeks. Criteria for continuation of treatment included stable clinical status, lack of disease progression, and the following laboratory parameters: absolute neutrophil count (ANC) ≥1000; platelets ≥100,000; urine protein ≤2+ on urinalysis; serum creatinine ≤1.5× upper limit of normal (ULN); aspartate aminotransferase (AST) serum glutamic oxaloacetic transaminase and total bilirubin ≤2.5× ULN; and as noted above, blood pressure consistently <140/90, and if elevated, controlled with antihypertensive medication(s).

Following completion of four cycles of therapy on this trial, surviving patients without PD continued treatment off trial, if tolerated, with standard concurrent chemoradiation with TMZ 75 mg/m^2^ daily and BV 10 mg/kg every 14 days. Patients who developed PD proceeded to standard chemoradiation without BV off trial.

### Response assessment

Radiographic assessment included noncontrasted and contrasted brain MRI every 4 weeks while on protocol. Baseline MRIs were performed within 2 weeks prior to starting treatment, and follow-up MRIs were performed at each cycle of therapy. At the time this trial was initiated, the Macdonald criteria were used for ongoing assessment of response. At each assessment, the sum of the products of the length and width of each lesion was calculated using its greatest diameter. The ratio of the sum at each follow-up time point and at baseline was calculated. If patients sustained clinical decline without radiographic progression, based on assessment from the treating practitioner at our institution, patients were removed from trial. For the purposes of radiographic assessment for this study and manuscript, all MRI scans from all time points were reviewed and interpreted by three neuro-oncologists (E. L., K. B. P., J. J. V.) according to published Radiologic Assessment in Neuro-Oncology (RANO) criteria 14, including Fluid attenuated inversion recovery (FLAIR) images. Increases in FLAIR were interpreted in context, and per RANO criteria were assessed as disease progression. Consensus was determined regarding assessment of response. Determination of response assessment was as follows: complete response (CR: ratio = 0), partial response (PR: 0 < ratio < 0.5), stable disease (SD: 0.5 < ratio < 1.25), or PD (ratio > 1.25). However, due to the limited timeframe of the study, some cases with radiographic PR as best response could not be confirmed with MRI 4 weeks afterward, per standard RANO criteria assessment. For the purposes of this study, the presence of multiple tumors was designated as “multifocal” if the individual tumors were ≥2 cm apart. In cases of equivocal radiographic progression with clear clinical decline of the patient, MRI assessment was designated as PD and patients were taken off trial.

### Statistical evaluation

A two-stage minimax study design was used to differentiate between response rates (CR + PR) of 10% and 26% assuming type I and II error rates of 10% 15. The patient population that was to be accrued to this study had a slightly worse prognosis than those treated with TMZ by Gilbert et al. 16, who reported a response rate of 42%, with a 95% confidence interval (CI) ranging between 26% and 59%. Therefore, we targeted a response rate with TMZ and Avastin that was on the low side of Gilbert's CI (i.e., 26%) as an indication that the combination would merit incorporation into a randomized phase III study. Kaplan–Meier methods were used to calculate survival estimates and plots for all 41 patients enrolled in this trial. Median survival was calculated from the start of study drug (cycle 1, day 1) to time of death or last contact if alive; as noted in Results and Discussion, this differs from standard OS reported in clinical trials, as our analysis included time of survival beyond enrollment on this trial.

## Results

Forty-one patients were enrolled in this study between October 2007 and September 2008 (Table 1). The regimen was tolerable for most patients, with 14 patients experiencing a severe, life-threatening, or lethal AE that was possibly, probably, or definitely related to treatment (Table 2). Twenty patients terminated treatment before completion of the 4-month treatment period, either because of clinical or radiographic progression (10 patients), death (three patients), or adverse experiences (six patients). One patient was lost to follow-up after three cycles of treatment. During the course of protocol treatment, three patient deaths occurred. Two of the three deaths that occurred were associated with clinical and radiographic progression, one within the first cycle and one within the second cycle, and were considered unrelated to the protocol treatment. The remaining death occurred within the second cycle and was attributed to a myocardial infarction, which was deemed possibly related. Prior to the patient's death, he had received four doses of BV. With regard to cardiac risk factors, the patient had a history of treated hypertension only, but during the protocol, the patient had documented hypertension that was associated with a grade 2 toxicity prior to the death.

**Table 1:** Patient characteristics

Characteristics	Number of patients
Total number of patients	41
Median age, years (range)	59.1 (40–75)
Sex	
Male	26
Female	15
Baseline KPS	
100	7
90	7
80	18
70	7
60	2
Unresectable status	
Multifocal	12
Single lesion	29
Surgery	
Biopsy only	41
Subtotal resection	0
Baseline steroids^1^	34
Completed cycles	
1^1^	9
2	8
3	3
4	21
Best response	
Partial response	10
Stable disease	28
Progressive disease	1
Unevaluable	2

KPS, Karnofsky performance status. Twenty-one patients completed all four planned cycles. Among these 21 patients, the final best response was partial response (PR) in 9/21 (43%) patients and stable disease (SD) in 12/21 (57%) patients. Among these 21 patients, four (19%) terminated after four cycles with progressive disease (PD). This number includes two patients who had a best response of a PR, and two patients who had a best response of a SD. The authors note that for the remaining 20 patients - i.e., those who only completed 1-3 cycles - the reason patients came off study was PD. For those with a best response of PR, this indicates that the patient was in PR when ending the study.

1 Dexamethasone.

2 One patient expired during cycle 1.

**Table 2:** Treatment-related toxicities

Toxicity	Number of patients	Toxicity grade
Hematologic		
Neutropenia	2	Grade 4
Thrombocytopenia	1	Grade 3
2	Grade 4
Venous thromboembolic	2	Grade 3
2	Grade 4
CNS hemorrhage	1	Grade 2
Bowel perforation	1	Grade 3
Impaired wound healing/infection at site of incision	1	Grade 3
Dehydration	1	Grade 3
Vomiting	1	Grade 3
Fatigue	2	Grade 3
1	Grade 4
Esophagitis	1	Grade 3
Transaminase elevation (AST)	1	Grade 3
Myocardial infarction	1	Grade 5

CNS, central nervous system; AST, aspartate aminotransferase.

**Figure 1:**
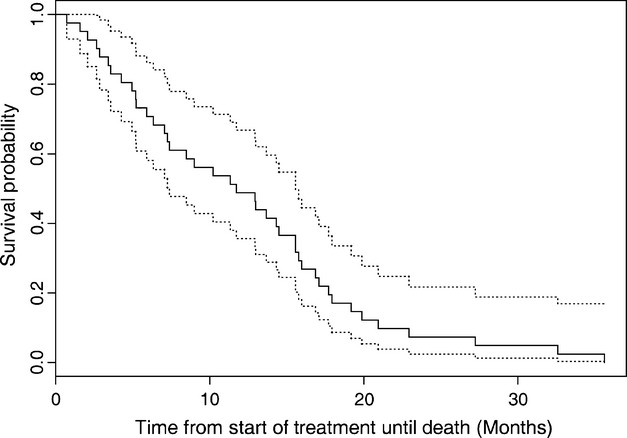
Kaplan–Meier plot for median survival from time of initiation of treatment on trial until death (including posttrial treatment and survival).

As enrollment and treatment on study was limited to a maximum of 4 months of upfront therapy, statistically accurate assessment of OS could not be made. We report here the median survival time from start of treatment until death measured in months (Table 3). Using this approach, median survival of the 41 enrolled patients was 11.7 months (95% CI: 7.4, 15.6 months) (Fig. 1).

**Table 3:** Survival estimates, reported as median time of survival from time of enrollment on trial until death. Number of enrolled patients (*n* = 41)

Survival	Months (95% CI)
Median survival in months (95% CI)	11.7 (7.4, 15.6)
6-month survival (95% CI)	70.7% (54.3, 82.2)
12-month survival (95% CI)	48.8% (32.9, 62.9)
24-month survival (95% CI)	7.3% (1.9, 17.8)

CI, confidence interval. Statistical analysis comprises survival data from salvage/poststudy therapy as well as planned treatment on trial for all 41 enrolled patients.

Thirty-nine patients had a complete set of images available for radiographic evaluation and measurements. Radiographic images could not be evaluated for the other two patients: one patient died during the first cycle due to clinical deterioration, and the other patient was lost to follow-up. Among the 41 patients, best overall response included 10 (24.4%) PRs, 28 (68.3%) SD, and 1 (2.4%) PD. We note that the RANO criteria specify that radiographic responses must be sustained for at least 4 weeks 14. Due to the 4-month limitation of the trial – which was initiated prior to changing from Macdonald to RANO criteria for assessing radiographic responses of GB – several of these patients did not meet strict technical RANO criteria for PR due to lack of available MRI images for review 4 weeks after PR was documented. Nonetheless, we included these patients in the PR category due to the significant radiographic regression of their tumors.

Twenty-one patients completed all four planned cycles, with 17 having at least SD after completion of the fourth cycle. Four of these 21 patients had documented radiographic PD following the fourth cycle, but had achieved at least SD as the best radiographic response during the course of the trial. Of 29 patients with single-lesion unresectable tumors, 11 completed four cycles without radiographic tumor progression. Figure S1 illustrates a typical radiographic response throughout the four cycles of therapy. Twelve patients were diagnosed with multifocal unresectable tumors, and of these, six patients completed all four cycles. Three of the 12 patients with multifocal unresectable GB showed signs of disease progression while on study; of note, the two patients on study who did not have evaluable MRIs beyond baseline had multifocal disease, but radiologic outcome could not be determined.

## Discussion

The optimal treatment of GB is surgical resection, followed by concurrent chemoradiation using TMZ therapy 1. Adjuvant therapy consists of 6–12 months of standard 5-day TMZ at 150–200 mg/m^2^ per 28-day cycle. Prognosis is better for patients who undergo gross total resection than for patients who undergo subtotal resection or who cannot undergo surgery due to tumor location 2–5. Despite the development of new therapeutics for the treatment of GB, patients with multifocal or single-lesions unresectable disease continue to have a much worse prognosis than patients with resectable disease. A newly published retrospective study further affirms the extent of poor prognosis in this patient population. The incidence of multifocal tumors was 12.8% (47/368 GB patients), with a median OS of 6 months, following biopsy and/or subtotal resection, followed by standard TMZ/radiation, as compared with 11-month OS for their comparator group of patients with single-focus disease. KPS was ≥70 in 76.6% of these patients with multifocal tumors 17.

This study found that neoadjuvant, or upfront, therapy with TMZ and BV resulted in a median survival time of 11.7 months in patients with unresectable disease. As noted by a reviewer, the limitation of this approach is that this differs from median OS, as the analysis included survival time of patients who received salvage/poststudy treatment following either radiologic progression or completion of four planned cycles on our trial. In a study by Stupp et al. 1, 573 patients with newly diagnosed GB were randomized to either standard radiation alone or concurrent chemoradiation with TMZ. Nearly 90% of these patients had WHO performance score of 0 or 1, similar to our study, in which median KPS was 80. Subgroup analysis of 93 patients with unresectable disease revealed a median OS of 7.9 months for radiation alone and 9.4 months for radiation plus TMZ 1. A larger scale, randomized trial encompassing upfront treatment with standardized concurrent chemoradiation therapy and beyond would be helpful in assessing whether or not survival benefit may be achieved with the addition of neoadjuvant BV/TMZ to standard radiation/TMZ in patients with unresectable GB.

Few published reports have specifically addressed the need to develop better treatments for patients with unresectable GB. A retrospective review of 50 patients with multifocal GB examined the impact of whole-brain radiation therapy (WBRT) versus three-dimensional (3D) conformal radiation. Patients had a mean age of 61 years; 71% of patients had a KPS >70%. Of patients, 32% were treated with WBRT; 68% had 3D conformal RT. The median time-to-progression (TTP) was 3.1 months, and median OS was 8.1 months. Local progression was seen in all patients. Multivariate analysis showed no difference in TTP or survival between either modality; thus, the recommendation was that WBRT not be mandatory in this situation 18. Another retrospective study addressing the appropriateness of aggressive treatment in elderly patients determined that among 206 elderly (>70 years of age; median age 75) patients with GB, the subpopulation with multifocal or multicentric disease had a significantly worse median OS (3.4 months, compared with 4.8 months for patients without multifocal disease). Other patients with significantly lower OS values included those with low KPS (≤50) or advanced age at time of initial diagnosis (≥80) 19. Our trial notably provides an upfront approach with chemotherapy and BV, in effect, delaying radiation treatment. This represents a new approach in treatment of GB, and the implications of this approach are as yet unknown. The rationale is both biologic and clinical. GB cells are known to readily secrete VEGF 20–22. Recurrent GB tumors secrete VEGF at a higher rate than newly diagnosed GB 22; thus, it is reasonable to consider the possibility that unresected tumors likewise secrete VEGF in excess at a higher rate than resected tumors. This consequent elevation of VEGF is associated with worse prognosis 23. Thus, these tumors should at least in theory benefit even more from VEGF inhibition upfront. Stabilization of tumor-associated vasculature by BV has been shown to improve tumor oxygenation and thus increase efficacy of ionizing radiation therapy of gliomas, as demonstrated in an orthotopic animal model of glioma 24. The unresected tumors develop hypoxia, which further stimulates the hypoxia-inducible factor1 alpha (HIF1-α) transcription factor and consequent VEGF secretion 25. A hypoxic tumor microenvironment would also lack oxygenation on which upfront radiation would depend for efficacy 24. One hypothesis is that achieving SD or PR with upfront therapy will improve outcomes of subsequent treatment with chemoradiation. A similar approach as our GB trial is currently being examined for resectable rectal cancer, for which the standard of care is neoadjuvant chemoradiation followed by surgical resection then further chemotherapy 26. This trial is assessing effects of chemotherapy alone with the purpose of determining if radiation can be safely delayed and even avoided altogether. For GB, statistically significant sub-analyses (such as in the trial by Stupp et al.) provide a historical comparison for biopsy-alone patients treated with upfront chemoradiation, and who have a worse prognosis than resected or partially resected patients.

The effects of BV on the natural biology of GB – and on many solid tumor malignancies – is not yet fully known, but is a topic of great interest. There is basic scientific evidence questioning whether BV may in fact increase GB cell invasion. However, these studies have largely been limited to in vitro and preclinical animal models, so further assessment is needed. The results of these studies should also be taken in context, such as short-term use of VEGFR inhibition 27. While VEGF inhibition with sunitinib of human-derived tumor spheroids in an in vivo model was found to decrease tumor enhancement while increasing proportion of infiltrating cells, the HIF1-α pathway is activated in the process. The authors suggest that vascular remodeling takes place and a more hypoxic tumor microenvironment predominates in the tumor model 28. In this case also, radiation would theoretically be less efficacious. Paez-Ribes et al. 29 performed a similar widely cited and intriguing study, which also includes examination of sunitinib on invasiveness of animal models of pancreatic neuroendocrine tumors and GB. However, the limitations of both major studies are pronounced by the single-agent approach using biologic VEGF inhibition 25,27,29, without the dual approach of standard chemotherapy treatment along with anti-VEGF treatment. Induction of hypoxia following VEGF inhibition again leads to activation of molecular pathways, which in all likelihood would be addressed by concurrent chemotherapy treatment. The importance of the upfront approach in our trial is in combining chemotherapy with biologic anti-VEGF therapy, for a predetermined amount of time (up to 4 months) in an already aggressive disease, in the hope that aggressive combination therapy upfront will enhance further treatment comprising radiation. Nonetheless, we are hopeful that this debate will be addressed by continued investigation at the basic science as well as clinical levels, to determine whether the potential benefits of BV are not outweighed by potential undesirable alterations to the natural cell biology of GB.

Regarding whether such treatment would alter required RT field size for further therapy, the potential for increasing RT ports should be small in light of an aggressive upfront treatment approach over 4 months. As noted above, at least one orthotopic xenograft model of GB demonstrated BV-induced stabilization of tumor vasculature, improved tumor oxygenation, and thus efficacy of ionizing radiation, suggesting benefit to this component of therapy 24. At least one trial on human patients – as phase II study of RSR13, an allosteric modifier of hemoglobin administered with intent to improve oxygen delivery during cranial irradiation for GB – demonstrated median OS >12 months. Seventy-eight percent of patients receiving RSR13 had been treated with resection; thus, 22% had biopsy alone and were included in these results 30. Nonetheless, further study is needed using in vivo animal models as well as subset analysis of future large-scale studies of patients enrolled in phase III trials using this approach. The results of one recent phase II trial from our group demonstrated increased OS when adding BV to standard TMZ/radiation followed by adjuvant treatment with TMZ/CPT-11(irinotecan)/BV for first-line treatment of GB 13. Another similar trial by Lai et al. 31 demonstrated an increase in PFS, but not OS, as compared with contemporary controls, when adding BV to standard chemoradiation. In both cases, the study authors called for additional, larger scale studies using BV in the first-line setting to make a more clear determination of benefit. It is reasonable to assume based on current available clinical data that the benefits of BV outweigh the potential risks, but clearly, further study is needed.

TMZ has been examined in the upfront setting in the elderly patient population. A retrospective analysis of a subset of patients from a randomized trial of radiation therapy versus supportive care alone for GB patients >70 years analyzed 39 patients who had declined either arm in favor of TMZ therapy alone; 21 (54%) of these 39 patients were considered unresectable and had biopsy alone, as compared with 36% who underwent subtotal resection and 10% who had complete tumor debulking. All patients were treated for up to 12 cycles with 5-day TMZ (mean number of cycles: 5). Responses included 1/39 CRs and 10/39 PRs. The median OS was 8.3 months for the whole group (as compared with 6.3 months for the 27 patients who did not receive second-line treatment at progression). The median PFS was 4.6 months for the whole group. The authors did not specifically analyze PFS and OS for the unresectable group. Significant toxicities included eight grade 3/4 toxicities: seven hematologic and one gastrointestinal 32. While the analysis for this study encompassed all patients (unresectable collectively with resectable), it is notable that the median PFS in our trial (100% unresectable GB) was higher, at 5.6 months, compared with 4.6 months in this prior study. As noted, the median age of patients in our study was 59.1 years, but there was a wide age range. A subsequent single-armed phase II study enrolled 70 elderly patients (median age 77) with GB treated with 5-day TMZ alone. Median PFS was 3.7 months, Median OS was 5.7 months (Table 4). Of these patients, 91.4% had biopsy-alone, and thus had unresected disease 33.

**Table 4:** Comparison of results of this study with prior trials or retrospective reviews, which analyzed the subset of patients with multifocal or single unresectable GB

Trial	RT alone 1	WBRT or 3D conformal RT alone 18	TMZ alone 32	TMZ alone 33	TMZ/RT 1	Variable (RT ± chemotherapy) 19	Upfront BV/TMZ [this study]
Median OS (months)	7.9	8.1^1^	8.3^2^	6.3^3^	9.4	3.4 months for multifocal; 2.8 months for unresectable	Median survival (including poststudy treatment) 11.7 (95% CI 7.4, 15.6)
Median age (years)	56 (entire study)	61	75	77 (entire study)	56 (entire study)	75	59.1
Number of patients	45	50	21	70	48	113	41
Type of trial	Phase II	Retrospective analysis	Retrospective analysis	Phase II	Phase II	Retrospective analysis	Phase II
Patient cohort examined	Subset of unresectable patients	Multifocal GB (unresectable and resectable)	Subset of patients with biopsy-alone treated only with upfront TMZ	Elderly patients, KPS <70; 91.4% of patients had unresected GB.	Subset of unresectable patients	Subset of patients with biopsy-alone and/or multifocal disease; variable treatment regimens	Unresectable single and multifocal GB

GB, glioblastoma; RT, radiation therapy; WBRT, whole-brain radiation therapy; 3D, three-dimensional; TMZ, temozolomide; BV, bevacizumab; OS, overall survival; KPS; Karnofsky performance scale; GTR, gross total resection; PFS, progression-free survival.

1 This number includes all patients reviewed; 22% of these patients had biopsy alone without attempt at surgical resection.

2 This number includes subset analysis of 39 patients who had declined RT in favor of TMZ alone following either biopsy or attempt at surgical resection; of those 39 patients, 21 (54%) had biopsy alone. The OS is for all 39 patients; no subset analysis was performed for the unresectable group.

3 Of the 70 patients enrolled in this phase II study, 64 (91.4%) had biopsy alone (i.e., unresectable GB); seven (7.2%) patients had partial resection prior to TMZ; one (1.4%) patient had GTR. PFS and OS as reported represent all enrolled patients.

Half of our enrolled patients completed four cycles of chemotherapy. We believe this is due to a combination of disease-related morbidity as well as potential adverse effects from therapy. Twenty-one patients completed all four planned treatments. Seventeen had SD after four cycles; four had PD following the fourth cycle, but had achieved at least SD as best radiographic response during the trial. We are encouraged by the fact that the TMZ/Bev combination given upfront was able to stabilize disease in these patients.

This combination was generally tolerable with toxicities consistent with prior studies evaluating these agents in the adjuvant setting with one potential study-related death due to a myocardial infarction. Screening for patients with comorbidities such as hypertension and hyperlipidemia should be considered when enrolling on clinic protocols involving BV. The degree of screening needed have yet to be determined and warrants further study and discussion. The risk of cardiac events in brain tumor patients receiving BV-containing regimens has not been evaluated, but there is evidence in the stage IV colorectal cancer population that BV-containing regimens do not increase cardiac events 34.

In conclusion, this study demonstrated the feasibility of administering TMZ and BV in the upfront setting to patients with multifocal or unresectable GB. The goal of this trial was to determine if there was enough biologic activity of TMZ with BV in the upfront setting to merit further investigation in larger scale randomized controlled trials, specifically for the unresectable population. We emphasize that patients with unresectable GB represent the subset with the worst prognosis of this disease. For this reason, it is not unexpected that a relatively low percentage of patients would have SD long enough to complete all four planned cycles. Upfront administration of TMZ and BV in this challenging patient population provided a significant level of disease stabilization. Our study was a single-institution trial with single-arm enrollment of 41 patients. Our study was limited by comparison to historical controls and changes in standard of care treatments since the publication of those prior trials, and lack of complete access to MRI scans following trial completion. PFS could not be assessed due to the following reason: as the study was designed for only four cycles, any data after the patient completed the four cycles were not collected prospectively, and thus would make PFS estimations less reliable. We were significantly limited by the poor prognosis of this patient population, in which subsequent MRI scans were not performed due to patient deaths. Among the 41 patients on this study, 17 experienced PD during the first four cycles of treatment. Among the remaining 24 patients, 13 had no MRI after the first four cycles. Among these 13 patients, the death date was >6 months after the off-study date in four patients, 3–6 months after the off-study date in five patients, and 0–2 months in four patients. Due to the limited timeframe of the study, and the aforementioned reasons already noted, accurate assessment of OS cannot be determined. Instead, we report median time of survival for all 41 patients, including all patients who could not complete four cycles of therapy; the limitation of this analysis is that it comprises posttrial treatment as well. Considering the limited subset analyses of this specific population (unresected GB) in other trials, it would be helpful to examine this regimen in larger, randomized clinical trials to more accurately address whether the biologic activity using this regimen in the upfront setting would result in improved OS and/or PFS.

A reviewer raised the issue of well-founded concerns in the oncology community regarding designs of appropriate trials for a malignancy that is relatively rare, and thus making it more difficult to provide large-scale randomized trials 35,36. The AVAglio study – a phase III trial of BV added to standard radiotherapy and TMZ for patients with newly diagnosed glioblastoma – is an excellent example of a well-designed randomized clinical trial designed to address the utility of VEGF inhibition in this malignancy 37. Chinot et al. recently presented results of their ongoing analysis of this trial, suggesting that the trial met its coprimary end point of improved PFS; interim analysis did not suggest significant increase in OS. The study enrolled patients with resectable as well as unresectable GB. Of patients enrolled on this trial, 42% had complete surgical resection prior to chemoradiation ±BV; 47% had partial resection. Distinction in the latter category between subtotal resection versus biopsy alone is not clear from the abstract, but if one is to presume that only 11% of the enrolled patients had unresected and/or multifocal disease, this represents a small portion of the total population. As noted, biologically, an intact unresected tumor may behave differently than the remaining tumor following surgery. While the potential benefits of BV in treatment of GB remain to be clarified at this point in time, as with other cancers, the use of biomarkers and subset analysis will help to identify subpopulations that are more likely to benefit from addition of BV, and that may in fact have an improved OS compared with other specific subpopulations. We eagerly await the matured data and analysis from the AVAglio trial, particularly the outcomes stratified by appropriate tumor markers.

We present our work as a starting point for discussion and consideration, with the hope that well-designed, larger scale phase III trials focusing on this unresectable GB population will provide accurate data for the utility of the upfront approach using BV. Our findings of biological activity of the TMZ/BV regimen in the upfront setting provide support for phase III randomized controlled trials investigating the use of this and other therapy combinations.

## Supporting Information

Figure S1 Representative MRI images showing radiographic response after four cycles of TMZ/BV therapy. MRI images are of a 71-year-old male patient with butterfly GB across the splenium of the corpus callosum. He was treated with a full four cycles of TMZ/BV on protocol. His tumor showed initial response to therapy, with the best response seen from baseline MRI (a) to post-cycle 2 MRI (b). His tumor then progressed during the fourth and final cycle (axial images with contrast are on the left; axial FLAIR on the right). MRI, magnetic resonance imaging; TMZ, temozolomide; BV, bevacizumab; GB, glioblastoma.
