# Supplementary figures and images for: Phase II trial of upfront bevacizumab and temozolomide for unresectable or multifocal glioblastoma
Source: Cancer Med. 2013 Jan 24;2(2):185–95. doi: 10.1002/cam4.58 (PMC3639657; doi:10.1002/cam4.58)

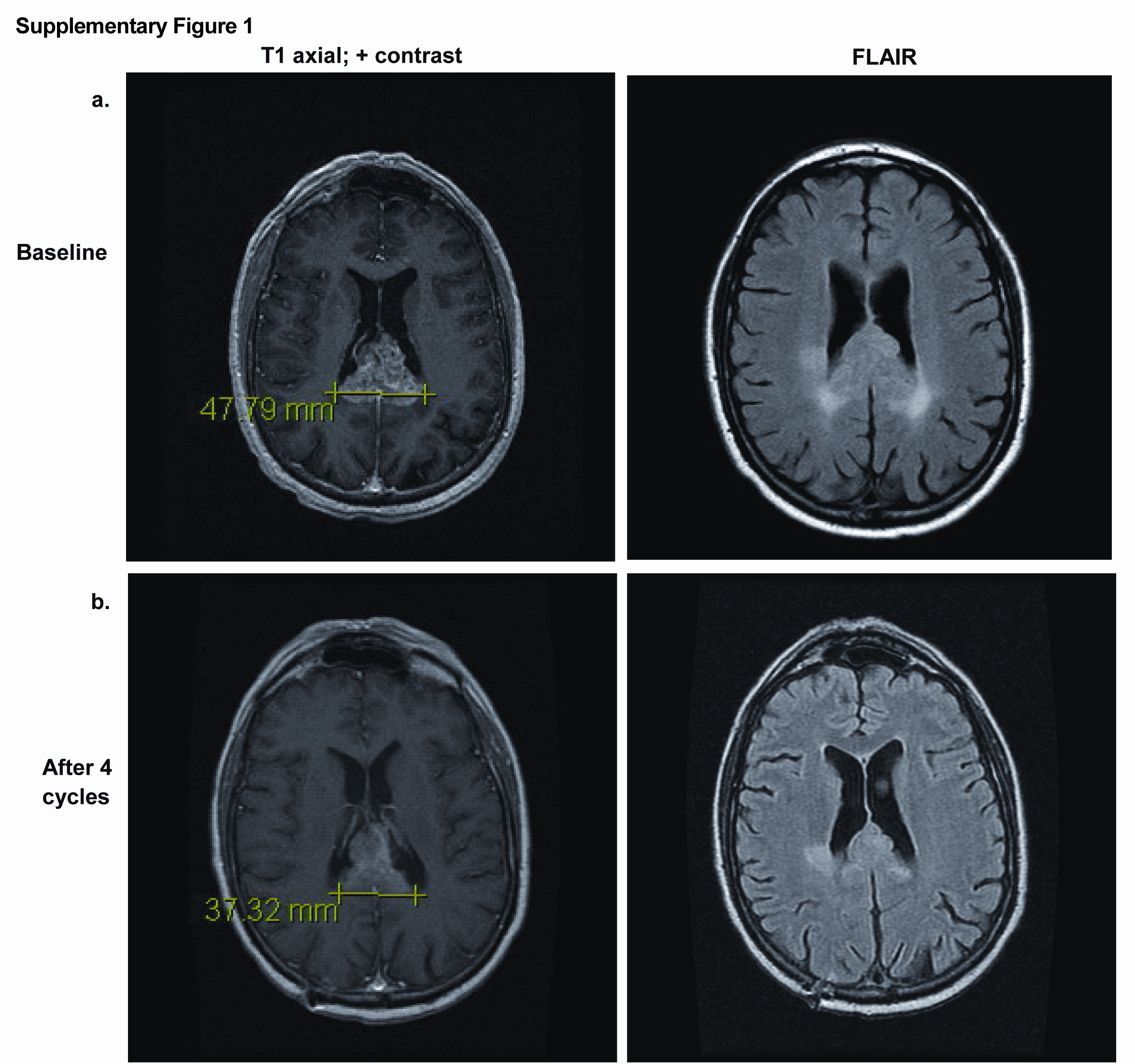

Supplement: Figure S1 — Representative MRI images showing radiographic response after four cycles of TMZ/BV therapy. MRI images are of a 71-year-old male patient with butterfly GB across the splenium of the corpus callosum. He was treated with a full four cycles of TMZ/BV on protocol. His tumor showed initial response to therapy, with the best response seen from baseline MRI (a) to post-cycle 2 MRI (b). His tumor then progressed during the fourth and final cycle (axial images with contrast are on the left; axial FLAIR on the right). MRI, magnetic resonance imaging; TMZ, temozolomide; BV, bevacizumab; GB, glioblastoma. [file cam40002-0185-sd1.tif]
